# Electrical Storms in Brugada Syndrome: Review of Pharmacologic and Ablative Therapeutic Options

**Published:** 2005-01-01

**Authors:** Maury P, Hocini M, Haïssaguerre M

**Affiliations:** * University Hospital Rangueil, Toulouse, France; † Hôpital Haut Lévèque, Pessac and University Victor Segalen Bordeaux II, France

## Abstract

Electrical storm occurring in a patient with the Brugada syndrome is an exceptional but malignant and potentially lethal event. Efficient therapeutic solutions should be known and urgently applied because of the inability of usual antiarrhythmic means in preventing multiple recurrences of ventricular arrhythmias. Isoproterenol should be immediately infused while oral quinidine should be further administrated when isoproterenol is not effective. In case of failure of these therapeutic options, ablation of the triggering ventricular ectopies should be attempted.

The Brugada syndrome is an inherited cardiac electrical disorder occurring in the absence of obvious structural heart disease, initially defined by the association of right bundle branch block, ST elevation in right precordial leads and sudden cardiac death related to polymorphic ventricular tachycardias [1].

In Brugada syndrome, the prevalence of malignant ventricular arrhythmias varies from 5 % at two years follow-up in asymptomatic patients to 45 % in patients with previous cardiac arrest [2]. While in most patients, a single arrhythmic event occurs, some patients will suffer electrical storms. Although there is no commonly accepted definition for this event, electrical storm is generally perceived as the rapid or incessant succession of recurrent poorly tolerated ventricular arrhythmias, generally requiring repeated cardioversions, occurring during a short period of time [3,4] (Figure 1). Electrical storm is fortunately a very scarce phenomenon, but which can occur twice or more in the same patient [5,6], or can be the first manifestation of the disease [7,8].

True prevalence of electrical storm in Brugada syndrome has not been evaluated, such events having only been seldom reported in isolated case reports. To our knowledge, to date, only twelve such cases have been published [5-16]..

Although exceptional, electrical storm in Brugada syndrome is a major event with dramatic consequences [7,10], leading sometimes to a fast ineluctable arrhythmic death in otherwise healthy young people despite all usual antiarrhythmic interventions [8]. Heart transplantation has even been once performed for such an intractable electrical storm [5]. That’s why some particular crucial and efficient therapeutic considerations should be known and urgently applied in order to avoid a fatal outcome.

## Pharmacological Management

Causal factors should be searched and immediately corrected, such as fever [8,15,16], increased vagal tone during gastro-enteritis [6], low potassium blood levels [17], non febrile  bronchitis [6] or infusion of class 1 drugs for diagnostic purposes [14,18,19].

Apart from class 1A agents (see further) no antiarrhythmic drug has been shown to be effective in preventing recurrence of arrhythmia in Brugada syndrome [20] [21], then antiarrhythmic drugs should be better avoided in case of electrical storm. Beta-blocker, bretylate, lidocaine, mexiletine or magnesium has been tried without any success [7,8,11,16] or can even worsen the situation [22]. In the DEBUT study, there was an 18 % death rate in Thai patients survivors of Sudden Unexpected Death Syndrome which were treated with beta-blocker [23]. If amiodarone infusion has been once apparently successful (but concommitantly with a beta-adrenergic agonist, see further) [7], it did not seem to be beneficial in other cases [8,12,16]. Sotalol, a drug devoid of class 1 effect, seems to have been successful in one case [7], however, one should remind that class 3 drugs also can increase ST elevation [18,24] that may be deleterious.

Isoproterenol infusion at a sufficiant dosing, ranging usually from 0.1 to 1-3 μg/mn [6,10,18,19], should be immediately started. Although isoproterenol is generally proarrhythmic in most other conditions, it is an effective drug in electrical storms related to Brugada syndrome and prevents recurrences of ventricular fibrillation (VF) [6,7,9-12,21].

Both Brugada electrocardiographic pattern and ventricular excitability or vulnerability are believed to be dependent on the sympathetic imbalance and on cardiac rate. Worsening of the electrophysiological conditions have been described when vagal tonus increases and/or heart rate decreases [22,25]. Major arrhythmic events and sudden death are known to frequently occur at night, when the vagal tone is predominant [21]. In patients with Brugada syndrome, isoproterenol infusion normalizes the electrocardiographic pattern and avoids ventricular fibrillation induction during electrophysiological study [9,11,21].

Beta-adrenergic stimulation with isoproterenol increases I_CaL_ and restores the dome of epicardial action potentials, reducing the degree of local and transmural heterogeneicity [21,26]. This can be sufficient to decrease the degree of ST elevation [22] and to avoid the genesis of premature beats. Beta-adrenergic stimulation should be optimally performed using isoproterenol, since failure of dobutamine infusion had been reported [8], although cilostazol - e.g. an oral phosphodiesterase inhibitor - has also been shown to be efficient in preventing recurrent VF in Brugada syndrome [27].

Accelerating the heart rate (decreasing I_to_) [28,29] or decreasing the vagal tonus (decreasing I_KAch_ and increasing I_CaL_) [26,29] for example by atropine infusion [28], can also act, particularly when associated to beta agonist [10]. However atropine infusion alone has been sometimes tried without any beneficial result [6], while success [30] or failure [8,9] of fast pacing have been reported.

In some cases, VF is incessant despite major adrenergic stimulation due to the physical and emotional stress caused by the repeated shocks. The associated alpha-adrenergic stimulation, which is leading to ST elevation in Brugada syndrome [21,29], is suspected to overwhelm the beneficial effects of beta-adrenergic stimulation in those cases [6].

Class 1A drugs like quinidine are another means to escape from such a critical situation and should be tested in patients with electrical storm not immediately responding to isoproterenol. Oral quinidine has been successfully used in electrical storms in Brugada syndrome [10,12,20].

Quinidine is a class 1 agent and should theoritically worsen the situation because of its sodium channel blocking properties. However quinidine is also a blocker of the transient outward current I_to_ [17,28,31]. Blocking I_to_ counteracts the marked abbreviation of action potentials in epicardial cells and so normalizes ECG pattern and ventricular vulnerability. Experimentally, class 1A drugs restore the action potential dome, normalize ST segment elevation and prevent arrhythmogenesis by blocking I_to_ [28]. Anticholinergic properties of class 1A agents would also contribute to this beneficial effect [20,28].

In 1987, Belhassen and coll. first document the ability of class 1A agents for prevention of inducibility in patients with idiopathic VF [20], which is believed to be caused in a large part by Brugada syndrome [18,21]. Long term beneficial action of quinidine has then been described in a population of idiopathic VF and Brugada syndrome [32]. In this study, lack of inducible arrhythmia under class 1A drugs (mainly quinidine) was possible in 80 % of patients with the Brugada syndrome and in all patients with idiopathic VF, and displayed good prognostic value since no recurrent arrhythmia could be documented after a mean follow-up of 9 years when patients were treated with class 1A agents (quinidine 1 to 2 g daily) [32]. In a recent publication of the same group, quinidine bisulfate at a mean dose of 1.5 g daily prevented inducibility in 88 % of patients with Brugada syndrome, without any recurrence of arrhythmia with a mean follow-up of 56 months [31]. Normalization of the ECG pattern and suppression of the ventricular premature beats as well as the induction of VF by quinidine (1 to 1.5 g daily) has also been described by other groups in isolated cases [12,17,33].

Hydroquinidine chlorydrate (600 to 900 mg daily) has also been recently shown to to normalize the ECG pattern in 34 % of patients with Brugada syndrome, to prevent arrhythmia inducibility in 76 % of asymptomatic patients, to prevent the occurrence of arrhythmic events in 90 % of those patients in which arrhythmia was rendered non inducible, and to avoid any recurrence of arrhythmia in implanted patients presenting with repeated shocks [34].

The effect of others class 1 A drugs is more controversial. Disopyramide, another blocker of Ito, is believed to be potent but in a lesser extent [20,28]: disopyramide can sometimes increase ST elevation [29] and may be proarrhythmic or antiarrhythmic according to the associated conditions [20]; in fact it has been shown to prevent inducibility while exagerating the ECG pattern in one case [35] and was inefficient in another case [11]. Procainamide increases ST elevation [29] and is proposed for the drug challenge for diagnosis of Brugada syndrome [21]; it failed to prevent VF induction and to normalize the ECG pattern in one case of idiopathic VF with right bundle branch, considered as a variant form of Brugada syndrome [33]. Other agents which block Ito without significant block of I_Na_, such as tedisamil, a drug currently in clinical trials for atrial fibrillation, could also be useful in this situation [20].

The only side effect would be an excessive QT prolongation. Because of the blocking action of class1A drugs on repolarizing K currents, these agents could unmask an associated LQT syndrome, since some congenital LQT syndrome and Brugada syndrome are both linked to mutations of the sodium channel and can coexist in the same patient [36]. Indeed, excessive QT prolongation in this context has been reported [12], although not observed by others [17].

Finally, in case of incessant VF recurring despite all these therapeutic options, general anesthesia has been sometimes performed with good results5, although failure has been reported [7,8].

## Non Pharmacological Management

Interventional therapy has been recently developed for treating electrical storm [37,38], and appears promising in such critical situations. Even if ventricular premature beats are rather infrequent in patients with Brugada syndrome [39], ICD-stored electrograms and ECG monitoring have shown that ventricular premature beats (VPB’s) precede spontaneous VF in the majority of cases and that they are identical to the one initiating the arrhythmias, which are always induced by the same premature beat in a given patient [40].

**Figure 2:**
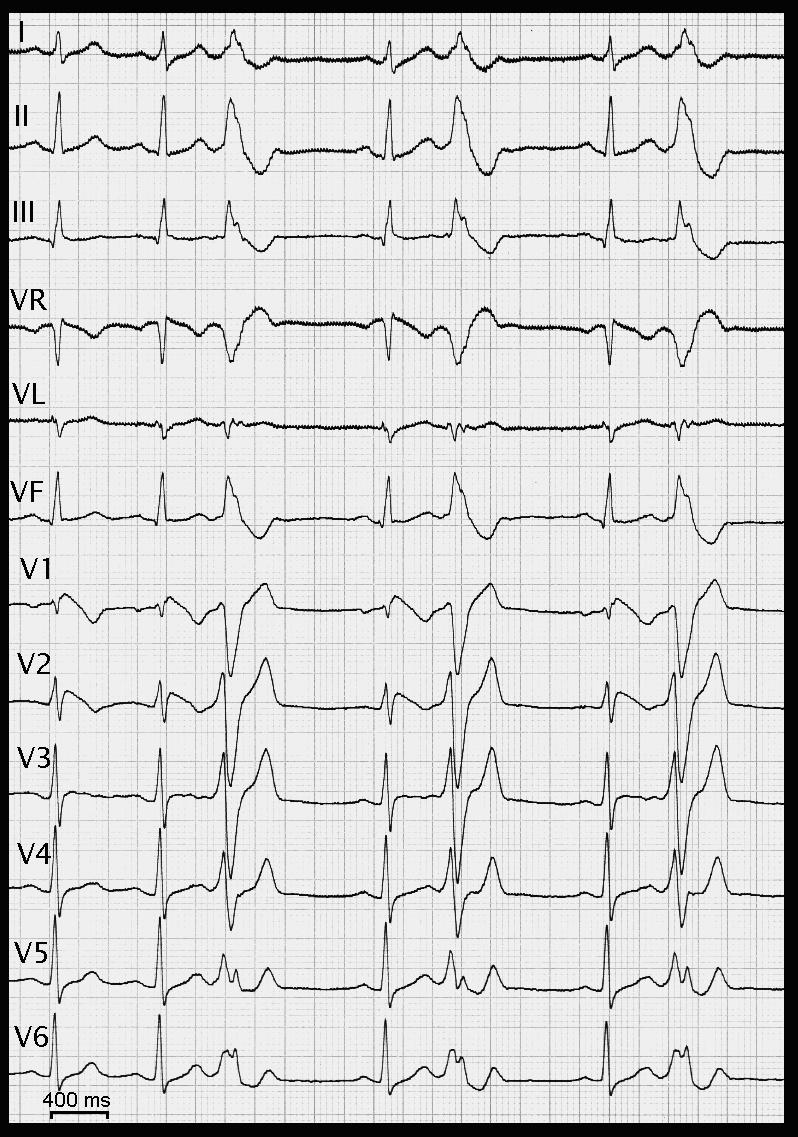
12 lead-ECG with typical ST elevation in right precordial leads and monomorphic ventricular bigeminy during drug challenge (Ajmaline infusion). These characteristic ventricular ectopies can be usually documented in Brugada syndrome, with left bundle block morphology and inferior axis, arising in the right ventricular outflow tract. These ectopies can trigger multi-recurrent arrhythmias and have to be targeted by radiofrequency ablation when interventional procedure is needed

Whether they induced VF or not, VPB’s in Brugada syndrome usually originate from the right ventricular outflow tract (RVOT) [12,;19,37] (Fig. 2), although right ventricular ectopics with left axis have been described [32,19,37,41]. Isolated cases of monomorphic ventricular tachycardia have been also reported, with left bundle block morphology with inferior [42,43] or superior axis [41,44,45] or even with right bundle block morphology [8,14,16], whose relationship with Brugada syndrome could be incidental or not, but which could also be incessant [14] and lead to fatal electrical storm [8]. Arrhythmogenic preponderance of the right ventricular outflow tract is not surprising due to the local higher electrical gradient, as otherwise ilustrated by the ST elevation which is usually exclusively present in right precordial leads.

Current observations suggest an important role for VPB’s of right ventricular origin in the Brugada syndrome. Chinushi et al. [46] described recurrent episodes of VF in a patient with Brugada syndrome initiated by monomorphic VPB’s with left bundle block morphology. This was corroborated by Morita et al. [47] who observed VPB’s in 9 out of 45 patients studied. Eleven VPB morphologies were observed in these 9 patients, of which 10 were of right ventricular origin (7 lateral RVOT, 2 septal RVOT and 1 from the apex).

While the cornerstone of the management of these conditions has been the implantation of a defibrillator, these reports and our recent success with the ablation of idiopathic VF [48] [49] has led us to evaluate the role of trigger elimination in patients with Brugada syndrome [37,50]. We have studied four patients with Brugada syndrome (3 males, age 36 ± 8 years). These patients presented with documented episodes of polymorphic ventricular tachycardia or VF (1 to 21 episodes), 3 with a family history of sudden death. They had 12 ± 9 episodes of VF or syncope prior to mapping. No drug therapy had been attempted in 3 patients with Brugada syndrome while quinidine failed in 1 patient. The Brugada syndrome was diagnosed by abnormal QRST complexes in leads V1 and V2 with a coved ST segment elevation in 4 patients, one who had a familial SCN5A channelopathy.  No patient had evidence of structural heart disease based on physical examination, echocardiography and right/left ventricular ejection fraction. Exercise testing and coronary angiography excluded myocardial ischemia. Brugada syndrome had been diagnosed 9 months and 3 years prior to the clinical episodes of VF in 2 patients.

All patients were studied within 2 weeks of their arrhythmic storm and had been documented to have frequent VPB’s. The triggering role of VPB’s in the initiation of VF was observed by ambulatory monitoring or stored electrograms of the defibrillator. Premature beats in the Brugada syndrome were monomorphic in all, coming from the RVOT in three patients (left bundle branch-inferior axis morphology, coupling interval of 343 ± 59 ms) and with left bundle branch block-superior axis morphology in one (coupling interval 278 ± 29 ms). The monomorphic VPB’s were first observed at the time of VF in 2 patients, whereas in 2 patients they had been documented together with a normal ECG 14 and 11 years before they triggered VF, preceding the apparition of ECG abnormalities. Exercise testing and isoproterenol infusion eliminated all premature beats, excluding catecholaminergic polymorphic ventricular tachycardia.

Mapping and ablation was performed as previously described in patients with idiopathic VF [49]. In the three patients with RVOT origin, VPB’s were eliminated by 7-10 minutes of radiofrequency energy applications at the earliest site of activity. In the fourth patient, the VPB’s were found to originate from the anterior right ventricular Purkinje network. Ten minutes of radiofrequency energy application eliminated all VPB’s in this patient. Noteworthy is that the inducibility of VF was modified after ablation. During a mean follow-up period of  9 ± 8 months there has been no recurrence of VF, syncope or sudden cardiac death in any patient.

While catheter ablation techniques are emerging, the initial experiences with idiopathic VF, and latterly with VF related to repolarization disorders have provided important insights into the role of focal triggers from RVOT and the Purkinje system in the initiation of VF associated with a number of clinical substrates in humans. Reducing the incidence of VF with localized ablation may reduce defibrillation requirement and replacement and improve the patients’ quality of life. The excellent long-term results after successful ablation of these triggers has been confirmed utilizing the data-logging capabilities of defibrillators in many of these patients and is being achieved in several centers.

## Figures and Tables

**Figure 1:**
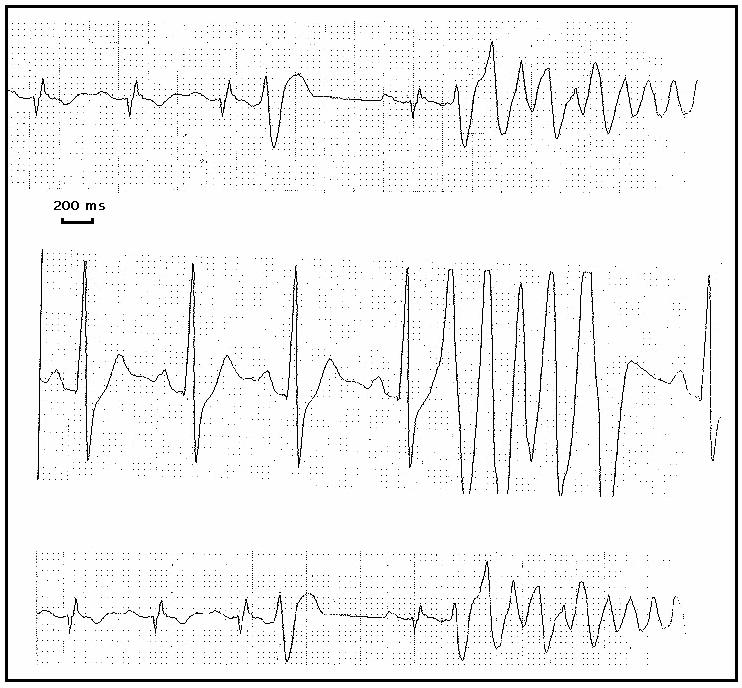
Repeated onset of polymorphic arrhythmias on ECG monitoring, as could be observed during electrical storm occurring in patients with Brugada syndrome
